# Retrospective study of gene signatures and prognostic value of m6A regulatory factor in non-small cell lung cancer using TCGA database and the verification of FTO

**DOI:** 10.18632/aging.103622

**Published:** 2020-09-09

**Authors:** Hongjie Shi, Jinping Zhao, Linzhi Han, Ming Xu, Kaijie Wang, Jiajun Shi, Zhe Dong

**Affiliations:** 1 Department of Thoracic and Cardiovascular Surgery, Zhongnan Hospital of Wuhan University, Wuhan 430071, China; 2 Department of Radiation and Medical Oncology, Zhongnan Hospital of Wuhan University, Wuhan 430071, China

**Keywords:** FTO, non-small cell lung cancer (NSCLC), prognosis, m6A, N6-methyladenosine

## Abstract

N6-methyladenosine (m6A) is the most common internal modification in eukaryotic mRNA. However, little is known about its role in non-small cell lung cancer (NSCLC). In this study, a total of 1017 NSCLC patients from the cancer genome atlas (TCGA) database with copy number variation (CNV) data were included. Log-rank tests and Cox regression model were used for survival analysis. The relationship between m6A regulators and clinicopathological features was evaluated using the chi-square test. The alteration of m6A regulators were related to T stage. Patients with any CNVs of regulators genes had worse overall survival (OS) than those with diploid genes. The deletion of m6A writer genes was an independent risk factor for poor OS, and the effect synergized with that of copy number gain of eraser genes. High expression of Fat mass-and obesity-associated gene (FTO) was associated with KRAS signaling up. Knockdown of FTO increased m6A content and inhibit proliferation of A549 lung cancer cell. Thus, we identified the genetic changes of m6A regulatory factors in NSCLC for the first time and found a significant relationship between these changes and poor clinical characteristics. FTO might play an important role in promoting NSCLC by decreasing m6A level and activating KRAS signaling.

## INTRODUCTION

Lung cancer is one of the most lethal tumors, with a mean survival rate of 18.4% at one year [1], and results in more than 1.3 million deaths per year [2]. NSCLC is the most common pathological type of lung cancer, accounting for about 85% of lung cancer [3]. In recent years, surgery, radiotherapy, chemotherapy, and targeted therapy have been shown to prolong the survival of patients with NSCLC [4]. However, the prognosis of NSCLC patients remains poor, with a 5-year survival rate of only 18% [5]. Therefore, there is an urgent need to find a new and meaningful biomarker or modification in NSCLC cells.

Apart from the genetic elements and proteins that play a vital effect in the occurrence and development of cancer, RNA modifications also play a crucial role in tumorigenesis. Among the RNA modifications, m6A is the most common, participating in multiple biological processes [6–8], such as cell death [9], cancer stem cell formation [10], tumorigenesis [11, 12], as well as contributing to the development of pathological conditions such as obesity and type 2 diabetes [13]. m6A occupies about 0.1–0.4% of the total adenosine residues in cellular RNA [6–8]. The process of m6A modification is mediated by enzymes that function as writers (methylases), erasers (demethylases), and readers [14]. Writers include METTL14, METTL3 and WTAP, and their complex promotes m6A modification in RNA. By contrast, erasers reverse the effect of writers in m6A-modified mRNA. FTO, the first recognized demethylase, has been proven to have cancer-promoting activity in gastric cancer, breast cancer, acute myeloid leukemia (AML), and cervical squamous cell carcinoma [15–19]. The readers enable the instructions of m6A modification to be converted into functional signals, including YTH domain family proteins [20].

In recent years, m6A regulators have been reported to enhance the development of diverse carcinomas, including liver cancer [12], acute myeloid leukemia [21], and glioblastoma [22]. However, m6A regulators also act as tumor suppressors in renal cell carcinoma [21]. Although many studies explain the role of m6A regulators in cancers, little is known about the function of m6A regulators in NSCLC. The present study aimed to understand the functions of m6A regulators in NSCLC by sequencing and analysis of CNV data from TCGA database.

## RESULTS

### Mutations and CNVs of m6A regulatory genes in NSCLC patients

**Figure 1:**
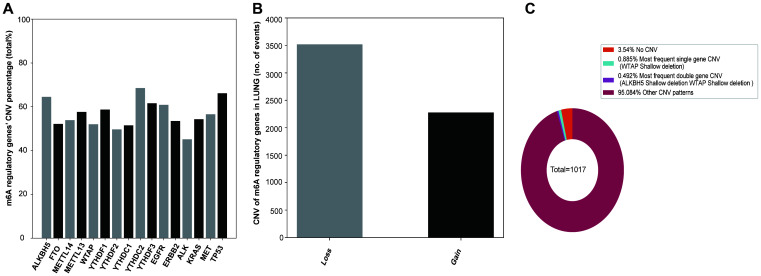
**CNVs of m6A regulatory genes in NSCLC.** (**A**) Percentage of lung cancer samples with CNVs in m6A regulatory factors in TCGA data. (**B**) Loss and gain of copy number of m6A regulatory factors in patients with NSCLC. (**C**) The most common CNV mutation pattern of m6A regulators in patients with NSCLC.

In the sequencing analysis, only 45 out of 408 samples showed mutations in the m6A regulatory genes (Supplementary Table 1). However, among the 1017 NSCLC samples with CNV data, CNVs were often observed in 10 m6A regulatory genes (Figure 1A). From these 10, the frequency of CNVs of YTHDC2 was the highest (68.53% 697/1017), that of YTHDF2 was the lowest (49.66% 505/1017), and that of the other 8 m6A regulatory factor genes was more than 50%. In addition, CNVs of TP53 and EGFR genes were higher in NSCLC patients, about 66.18% and 60.89%, respectively than in controls.

Next, we explored the CNV mutation pattern of m6A regulatory factors in NSCLC patients and found that the most frequent CNV type was loss of copy number, and the frequency was similar to that in ccRCC [23] and AML [24] (Figure 1B, Table 1). Because of the high frequency of CNVs of m6A regulatory factors in NSCLC patients, the frequency of CNV of only one regulatory factor or that of two genes at the same time is relatively small. The results showed that the shallow deletion of WTAP is the most frequent CNV of m6A regulatory genes (0.885%) and shallow deletion of WTAP and copy number gain of YTHDF3 were the most frequent double-gene CNV (0.492%) (Figure 1C).

**Table 1:** Different CNV patterns found in lung cancer samples (n = 1017).

	**gene**	**Diploid**	**Deep deletion**	**Shallow deletion**	**Copy number gain**	**Amplification**	**CNV sum**	**Percentage**
**Eraser**	ALKBH5	361	9	510	131	6	656	64.50%
	FTO	487	7	324	193	6	530	52.11%
**Writer**	METTL14	469	0	473	75	0	548	53.88%
	METTL3	431	4	270	296	16	586	57.62%
	WTAP	488	8	382	138	1	529	52.02%
**Reader**	YTHDF1	420	1	106	458	32	597	58.70%
	YTHDF2	512	4	332	166	3	505	49.66%
	YTHDC1	493	2	374	124	24	524	51.52%
	YTHDC2	320	8	585	102	2	697	68.53%
	YTHDF3	391	3	117	474	32	626	61.55%
**Hot gene**	EGFR	398	6	85	465	63	619	60.87%
	ERBB2	473	1	148	370	25	544	53.49%
	ALK	558	5	46	397	11	459	45.13%
	KRAS	465	2	128	366	56	552	54.28%
	MET	442	5	130	416	24	575	56.54%
	TP53	344	10	577	85	1	673	66.18%

### Relationship between alterations in m6A regulatory factors and clinicopathological and molecular characteristics

We evaluated the relationship between alterations in m6A regulatory genes (CNV and mutation) and clinicopathological parameters of patients. The results showed that there was a significant correlation between the change in m6A regulatory factors and T stage (p= 0.02) (Table 2). Next, we evaluated the relationship between m6A regulatory gene alterations and the hot genes (EGFR, ERBB2, ALK, MET, TP53 and KRAS) in NSCLC. As expected, there was a significant relationship between the alterations in m6A regulatory factors and alterations in these six genes. The result indicated the patients with mutation and CNV had more the alterations of hot genes than the patients without mutation or CNV (p<0.0001) (Table 3).

**Table 2:** Clinicopathological parameters of NSCLC patients with or without mutation/CNV of m6A regulatory genes.

		With mutation and/or CNV*	Without mutation and CNV*	**P-value**
**Age**	<=60	690	7	0.417
	>60	255	29	
**gender**	Female	372	18	0.269
	Male	573	18	
**Pathological Stage**	I	479	22	0.363
	II	267	10	
	III	157	3	
	IV	32	0	
	Discrepancy	10	1	
**T stage**	T1	259	18	0.02
	T2	536	11	
	T3	107	6	
	T4	41	1	
	TX	2	0	
**N stage**	N0	600	30	0.162
	N1	218	3	
	N2	105	3	
	N3	7	0	
	Nx	15	0	
**M stage**	M0	709	23	0.078
	M1	204	0	
	MX	32	13	

*With mutation or CNV: Cases have mutant or CNV or mutant+CNV, confirmed through TCGA database. Without mutant and CNV: Cases with neither mutant nor CNV, confirmed through TCGA database. Ambiguous variables (Nx, Mx, N/A, discrepancy and Gx) were excluded from chi-square test or non-parametric test.

**Table 3:** Relationship between molecular characteristics and alteration of m6A regulatory genes in patients with NSCLC.

			Without mutation or CNV*	With mutation and CNV*	***X*^2^**	**P**
**EGFR**	N=1017	wt	4	615	36.65	<0.0001
		alteration	32	366		
**ERBB2**	N=1017	wt	0	544	40.72	<0.0001
		alteration	36	437		
**ALK**	N=1017	wt	0	459	28.84	<0.0001
		alteration	36	522		
**KRAS**	N=1017	wt	5	547	22.87	<0.0001
		alteration	31	434		
**MET**	N=1017	wt	2	573	37.36	<0.0001
		alteration	34	408		
**TP53**	N=1017	wt	1	672	64.11	<0.0001
		alteration	35	309		

*With mutation or CNV: Cases have mutant or CNV or mutant+CNV of m6A regulators, confirmed through TCGA database. Without mutant and CNV: Cases with neither mutant nor CNV of m6A regulators, confirmed through TCGA database.

**Figure 2:**
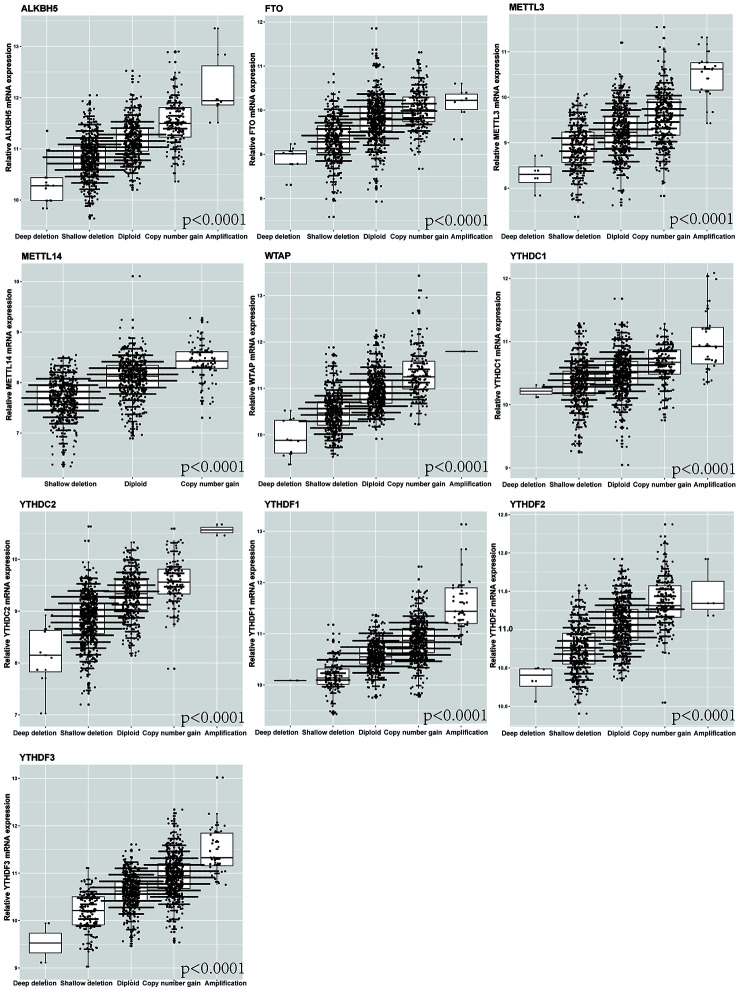
**Correlation between different CNV patterns of 10 m6A regulatory genes and mRNA expression level.**

Furthermore, we detected the effect of the alterations of m6A regulatory factors on mRNA expression in NSCLC patients. The results revealed that mRNA expression was significantly correlated with various CNV mutation patterns (p < 0.0001). For all the top 10 genes with CNV, copy number gain was associated with increased mRNA expression, while copy number loss was associated with decreased mRNA expression (Figure 2).

### Relationship between CNVs of m6A regulatory genes and survival of NSCLC patients

**Figure 3:**
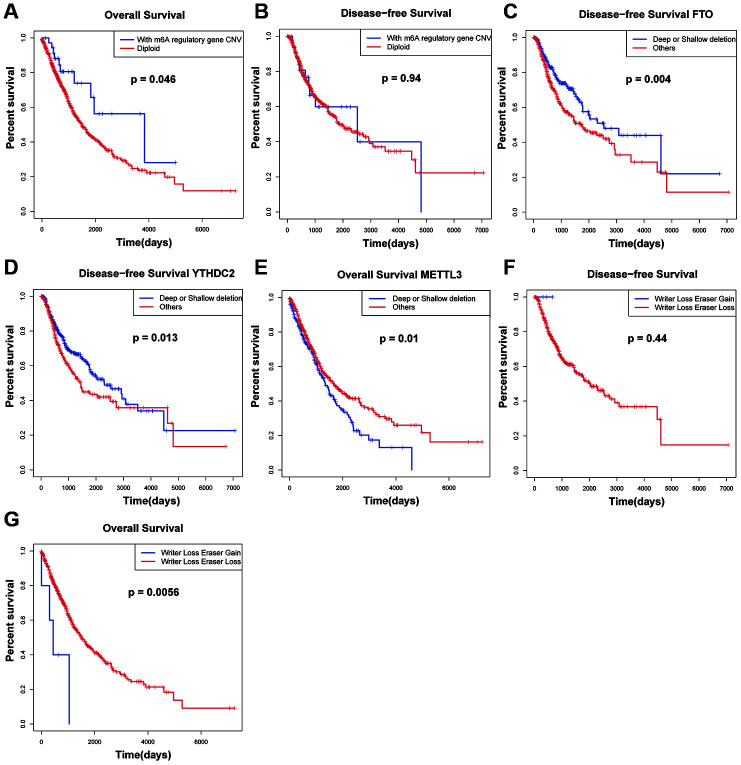
**Survival rate of patients with CNVs of m6A regulatory factors.** (**A**, **B**) Relationship between OS, RFS and m6A regulator carrying CNV or diploid in NSCLC patients. (**C**, **D**) DFS for NSCLC patients with different CNV patterns of FTO *and* YTHDC2. (**E**) OS for NSCLC patients with different CNV patterns of FTO *and* YTHDC2. (**F**, **G**) Relationship between simultaneous changes in m6A regulatory factors: writer genes and eraser genes, and OS, and DFS in NSCLC patients.

We also analyzed the relationship between CNVs of m6A regulatory genes and OS and DFS of patients. The results showed that the patients with m6A regulatory gene CNV had better OS than the patients with diploid. (Figure 3A and Figure 3B). Then, the OS and DFS analysis with respect to 10 m6A regulatory genes in NSCLC patients were carried out. The results showed that the patients with FTO and YTHDC2 deletion CNVs had better DFS than those with diploid and copy number gain (Figure 3C and Figure 3D) and patients with METTL3 deletion CNVs had worse OS than those with diploid and copy number gain (Figure 3E). The other genes showed no significant effects. Multivariate Cox regression analysis showed that the alteration of m6A regulatory genes was an independent risk factor for poor OS (Table 4). In addition, the "writers" are a group of methyltransferase genes, and are the most important part of the regulation process of m6A. "Erasers" are a group of demethylase methyltransferase genes. The results show that the "writer" gene down-regulation and "eraser" gene up-regulation may lead to the decline of survival rate of patients.

**Table 4:** Clinical Information and risk model univariate/multivariate COX analysis of m6A regulatory genes for overall survival and disease-free survival of patients with NSCLC.

**Variables**	**OS**				**DFS**			
	**Univariate analysis**		**Multivariate analysis**		**Univariate analysis**		**Multivariate analysis**	
	**HR(95%CI)**	**p.Value**	**HR(95%CI)**	**p.Value**	**HR(95%CI)**	**p.Value**	**HR(95%CI)**	**p.Value**
**Age**(≥60 vs.<60)	1.21(0.92-1.57)	0.168			1.04(0.75-1.45)	0.798		
**Gender** (male vs female)	1.23(0.97-1.55)	0.089			1.03(0.77-1.38)	0.835		
**Pathologic stage** (I-II vs III + IV)	1.86(1.46-2.38)	<0.0001*	1.19(0.83-1.7)	0.348	1.89(1.35-2.63)	<0.0001*	1.28(0.82-1.99)	0.269
**M** (M1 vs M0)	1.98(1.21-3.24)	0.006*	1.64(0.95-2.83)	0.078	1.37(0.61-3.1)	0.449		
**N** (N1, N2, N3 vs N0)	1.53(1.23-1.91)	<0.0001*	1.38(1.07-1.79)	0.014*	1.65(1.24-2.2)	0.001*	1.51(1.08-2.11)	0.015*
**T** (T3-T4 vs T1-T2)	1.74(1.33-2.29)	<0.0001*	1.55(1.11-2.15)	0.01*	1.89(1.32-2.73)	0.001*	1.64(1.06-2.53)	0.026*
**EGFR** (altered vs diploid)	1.18(0.94-1.48)	0.148			1.3(0.98-1.74)	0.071		
**ERBB2** (altered vs diploid)	0.94(0.76-1.18)	0.614			1.09(0.82-1.45)	0.549		
**ALK** (altered vs diploid)	1.27(1.02-1.59)	0.035*	1.35(1.07-1.69)	0.01*	1(0.75-1.33)	0.984		
**KRAS** (altered vs diploid)	0.91(0.73-1.13)	0.391			0.9(0.67-1.19)	0.451		
**MET** (altered vs diploid)	1.17(0.94-1.47)	0.156			1.34(1.01-1.78)	0.045*	1.31(0.97-1.75)	0.075
**TP53** (altered vs diploid)	0.96(0.76-1.22)	0.74			1.06(0.78-1.43)	0.719		
**m6A regulator alteration** (Writer Loss + Eraser Gain vs others)	0.27(0.1-0.73)	0.01*	0.31(0.11-0.85)	0.022*	1212451(0-Inf)	0.992		

Ambiguous variables (Nx, Mx, N/A, discrepancy and Gx) were excluded

In order to verify our results, we tested the association between CNVs combinations with different patterns of m6A carried by patients and OS and DFS. We found that compared with patients with only writer gene deletion, when the down-regulation of "writer" genes and the up-regulation of "erasers" genes occur simultaneously, the prognosis of patients was worse (Figure 3F and Figure 3G).

### Enrichment analysis of “eraser” genes: FTO

**Figure 4:**
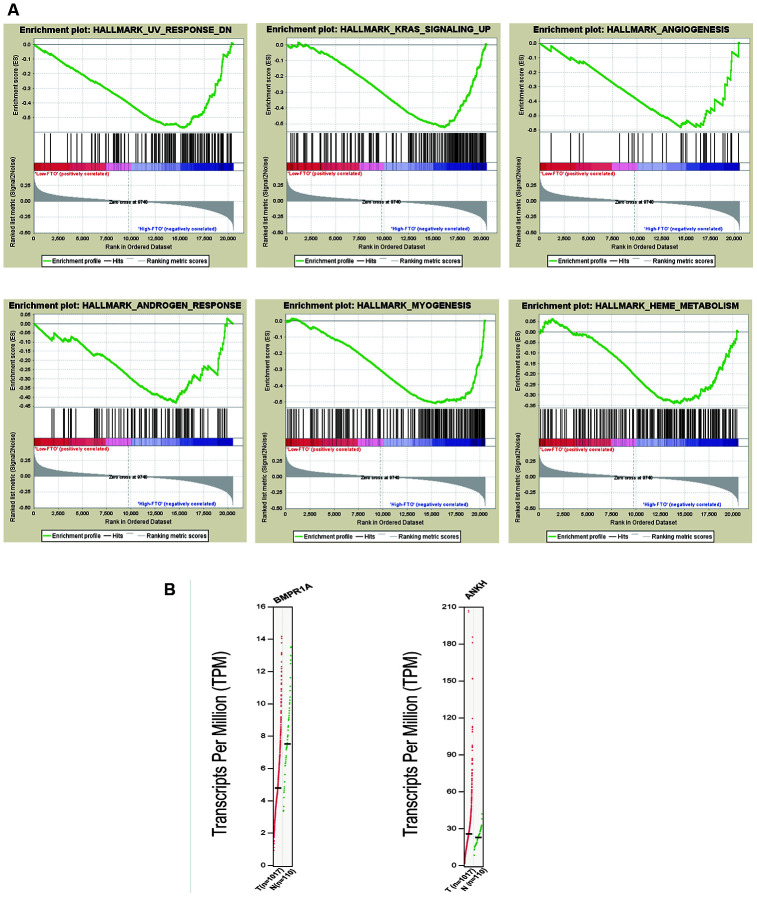
**Enrichment analysis of “eraser” genes: FTO.** (**A**) GSEA results of FTO with different expression levels. (**B**) Expression of genes related to enrichment pathway.

It has been shown that FTO is related to the occurrence and development of NSCLC. Thus, we divided all samples into high and low FTO mRNA expression levels according to the median FTO mRNA expression levels and performed the GSEA to conduct enrichment analysis. The high expression of FTO was significantly enriched in nine related pathways such as UV radiation response, myogenesis, KRAS signaling pathway and TGF-beta signaling (Figure 4A and Supplementary Table 2). Therefore, the genetic alterations of m6A regulatory factors in NSCLC were related to poor survival. To test our findings, we examined gene expression associated with these pathways. The result demonstrated that BMPR1A associated with TGF-beta signal and UV radiation pathway and was downregulated in NSCLC tissues. ANKH associated with androgen response pathway was upregulated in NSCLC tissues (Figure 4B). Besides, several studies have found that FTO could participate in UV radiation response, myogenesis and androgen response, which are consistent with our results [25–27].

### FTO inhibition suppressed the proliferation of NSCLC cells and Increase the level of mRNA m6A in A549

**Figure 5:**
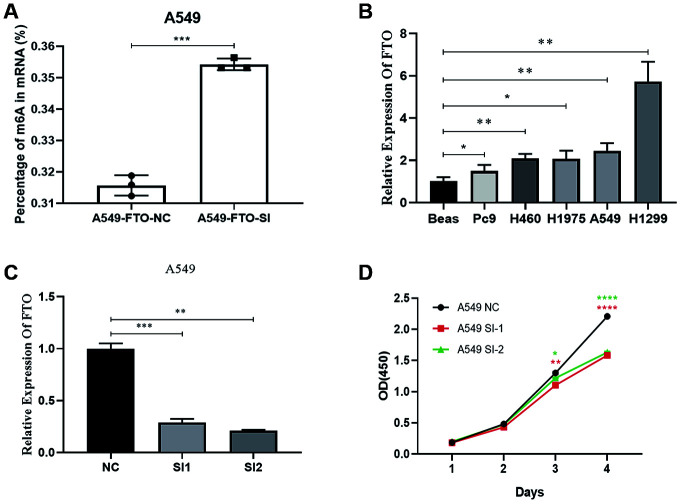
**Effects of silencing FTO on proliferation and mRNA m6A level of lung cancer cells.** (**A**) The mRNA m6A level in human lung cancer cell. (**B**) The expression of FTO in lung cancer cells. (**C**) Verification of knockout efficiency. (**D**) Knockdown of proliferation capacity of FTO inhibited lung cancer cells. (*, p < 0.05; **, p < 0.01; ***, ****, p < 0.001).

By the EpiQuik m6A RNA Methylation Quantification Kit, we found the m6A content in A549 knocked down the FTO increased (Figure 5A). Furthermore, using qRT-PCR, we found that FTO was highly expressed in A549. Then we used silencer-FTO to knock down the FTO (Figure 5B and Figure 5C). Next, by the CCK-8 assay, we showed knockdown of FTO inhibited the proliferation capacity of A549 lung cancer cell (Figure 5D). This is consistent with our results.

## DISCUSSION

N6-methyladenosine (m6A) is the most common internal modification in eukaryotic mRNA, the abundance of which varies from 0.1% to 0.4% of total adenosine residues [6–9]. Actually, previous study proved m6A existed in mRNA of more than 7600 genes and over 300 non-coding RNA [28]. It has been reported that m6A is involved in a variety of cellular processes such as cell proliferation, self-renewal, development and cell death [9]. Considering the importance of m6A in transcriptome, some studies have attempted to reveal the role of m6A in cancer and the results shown the regulators of m6A exert enormous functions in cancer development, such as proliferation, migration and invasion [29, 30]. In recent years, m6A regulators have been reported to enhance the development of diverse carcinomas, including liver cancer (11), acute myeloid leukemia [21], and glioblastoma [22]. However, m6A regulators also acted as tumor suppressors in renal cell carcinoma [31]. In our study, the frequency of alterations of the ten m6A related genes was much higher than that shown in AML and ccRCC, suggesting that dysregulation of m6A may play a significant role in the occurrence and development of NSCLC than AML and ccRCC [23, 24]. Thus, having a better understanding and further exploring the underlying mechanism for the m6A regulators in NSCLC is necessary.

Based on the statistics of 408 NSCLC patients, we found the mutations related to m6A regulatory factors in 45 samples. From the 10 m6A regulatory genes, shallow deletion of m6A “writer” gene WTAP and the copy number gain of m6A “reader” gene YTHDF3 were the most frequent double gene alterations, and shallow deletion of m6A “writer” gene WTAP was the most frequent single alteration, implying the importance of m6A writer genes in the process of RNA m6A modification. Due to the fact that EGFR, ERBB2, ALK, MET, TP53 and KRAS played important roles in the pathogenesis of NSCLC, we further evaluated the correlation between m6A regulatory gene variation and the alterations of six hot genes in NSCLC. The result indicated the patients with mutation and CNV of m6A regulators had more the alterations of hot genes than the patients without mutation or CNV (p<0.0001). Previous studies shown that EGFR mutation was related to the level of mRNA methylation in pancreatic cancer [32], and TP53 mutation was related to the level of mRNA methylation in gastric cancer [33].

Upon analyzing the relationship between CNV/mutation in m6A regulators and clinicopathological parameters of patients by chi-squared test, we found that there was a significant correlation between the change of m6A regulatory factors and T stage (p=0.02). Multivariate Cox regression analysis showed that the alteration of m6A regulatory genes was an independent risk factor for poor OS. For all the top 10 genes with CNV, copy number gain was associated with increased mRNA expression, while copy number loss was associated with decreased mRNA expression, revealing that mRNA expression was significantly correlated with various CNV mutation patterns. Besides, the results showed that the patients with m6A regulatory gene CNV had better OS than the patients with diploid. Sun et al. found that NSCLC patients with high expression of METTL3 was associated with better OS [34]. However, Gregory et al. indicated the upregulation of METTL3 could promote the growth, survival, and invasion of human lung cancer cells [35]. In the present study, patients with METTL3 (writer gene) deletion CNVs had worse OS than those with diploid and copy number gain. Moreover, the patients with FTO (eraser gene) and YTHDC2 (reader gene) deletion CNVs had better DFS than those with diploid and copy number gain. Previous study suggested lung cancer patients with depletion of FTO had better prognosis [36]. These results suggested that the down-regulation of m6A level might be associated with poor patient survival. Next, to validate our result, the associations among CNVs combinations with different patterns of m6A carried by patients and OS and DFS were tested, and we found that the "writer" gene down-regulation and "eraser" gene up-regulation may lead to the decline of survival rate of patients. This was consistent with our results.

Furthermore, we found that some studies have discussed the possible mechanisms of action of METTL3 in NSCLC. For example, METTL3 contributes to transforming growth factor-beta-induced epithelial-mesenchymal transition of NSCLC cells through the regulation of JUNB [37]. METTL3 has been found to promote protein translation of oncogenes in lung cancer cells through methyltransferase-independent activity [38]. However, little was known about the mechanisms of action of FTO. Thus, we further explored the correlated pathways with FTO. FTO was originally identified as a fat mass and obesity-associated protein and has been regarded as the first RNA demethylase in recent study [15, 39]. The GSEA analysis suggested that the high FTO expression was significantly enriched in UV radiation response, angiogenesis and KRAS signaling. Moreover, we found that BMPR1A related to TGF-β signaling and UV radiation response was down-regulated in NSCLC tissues and ANKH related to androgen response was up-regulated, which partially validate the GSEA results. OI. Kit et al. analyzed the copy number variation of some gene loci in lung tumor cells extracted by laser capture microdissection and in cell-free DNA in the plasma of patients with lung adenocarcinoma and detected the copy number variation of KRAS and FTO at the same time [40]. GSEA in high expression of FTO showed enrichment of genes up-regulated in KRAS signaling, indicating the activation of KRAS signaling by the upregulation of FTO. In addition, the results have indicated the patients with mutation and CNV had more KRAS alteration than the patients without mutation or CNV (p<0.0001), suggesting there was a significant correlation between KRAS alteration and m6A regulatory gene alterations. KRAS was the most frequently mutated oncogene in NSCLC and KRAS mutations were often associated with low overall survival and resistance to treatment [41, 42]. Previous studies have shown that MEK-ERK depletion reduced the expression of EZH2 in cells with KRAS (G12C) mutation, thereby reducing the level of histone methylation and inhibiting the development of cancer [43]. Therefore, we thought that the eraser gene FTO might down-regulate the m6A level of key molecules in KRAS signaling and further activate KRAS signaling to promote tumor.

Additionally, we found FTO was highly expressed in NSCLC cell A549 and knocking down FTO could inhibit the proliferation of A549, and mRNA m6A content analysis showed that the m6A content in A549 that knocked down the FTO increased. All this partly proved our hypothesis: the down-regulation of m6A level might be associated with poor survival in NSCLC patients. FTO has been reported to participate in the development of NSCLC. Depletion of FTO inhibited the proliferation, invasion, emigration of lung cancer cells [36]. And research has suggested that FTO facilitates lung adenocarcinoma cell progression by activating cell migration through m6A demethylation [44].

In summary, we determined, for the first time, the genetic alterations in m6A regulatory genes in NSCLC and identified a significant relationship between the alterations resulting in decreased m6A level and worse clinical characteristics including survival. Moreover, we found eraser gene FTO might play an important role in promoting NSCLC by decreasing m6A level and activating KRAS signaling. Future studies uncovering the oncogenesis mechanisms of FTO will be required to confirm our findings.

## MATERIALS AND METHODS

### NSCLC TCGA data

NSCLC-related data, including somatic non-silent mutation (gene-level), phenotype, gene expression, RNA-seq, and copy number (gene-level) data, were downloaded from TCGA (https://cancergenome.nih.gov/).

### Mutation of m6A regulatory genes and characterization of CNV in NSCLC patients

The somatic mutation data were used to calculate the somatic mutations of m6A regulatory factors and the copy number (gene level) data were used to calculate the CNV mutation pattern distribution of m6A-related regulatory factors in NSCLC patients. We collected the mutation information of the patients and selected for information of m6A gene. Simultaneously, statistical analyses and visualization were performed to assess the percentage of NSCLC samples with CNV in all patients, the number of samples with amplification and deletion, and the frequency of one or two regulatory factors with CNV.

### Analysis of the relationship between the changes in m6A regulatory factors and clinicopathological and molecular characteristics of tumor patients

COX regression analysis was used to explore the correlation between different CNV patterns and the levels of m6A regulatory gene mRNA. The relationship between CNV of m6A-related regulatory factors and clinicopathology was evaluated. Chi-square test was used for analyzing statistical significance.

### Association between CNV of m6A regulatory genes and survival of cancer patients

The effects of CNVs on overall survival (OS) and disease-free survival (DFS) of cancer patients and the risk factors were assessed by survival analysis and a univariate/multivariate Cox regression model.

### Enrichment analysis of m6A regulatory genes

GSEA was performed to enrich and analyze m6A regulatory genes and the genes that affected cellular pathways were selected.

### Quantitative reverse transcription PCR (qRT-PCR) and mRNA m6A level in human NSCLC cells

Total RNA was isolated from cells with RNA Extraction Kit (Aidlab Biotechnologies Co, Ltd, China). The RNA concentrations were detected using a Nanodrop spectrophotometer (Thermo Scientific Ltd., USA). cDNA synthesis was completed using a reverse transcription kit according to the manufacturer’s instructions. qRT-PCR was performed on the CFX Connect^TM^ Real-Time PCR Detection System (Bio-Rad, USA) using the ChamQ^TM^ SYBR® qPCR Master Mix. The expression of GAPDH was used as the reference for normalization. The 2^-ΔΔCt^ method was used to calculate the relative fold change in mRNA expression. The EpiQuik m6A RNA Methylation Quantification Kit (Colorimetric) (P-9005, Epigentek, USA) was used to measure the m6A content in total RNAs.

### Cell proliferation assay

Cell proliferation was assessed using the Cell Counting Kit-8 (CCK-8; Beyotime, Hangzhou, People’s Republic of China) according to the manufacturer’s instructions. Transfected A549 cells (3000 cells/well) were seeded in 96-well plates with three replicate wells per group. The optical density (OD) was measured at 450 nm every 24 hours using a multimodal plate reader (PE Enspire, USA).

### Statistical analysis

The R software was used to analyze all statistics and graphs. Chi-square test was used to analyze the association between m6A regulatory gene CNVs and clinicopathological features. Kaplan-Meier curve analysis and the logarithmic rank test were used to evaluate the predictive value of changes in m6A regulatory genes. Survminer and survival packages in R language were employed for Cox proportional hazard regression model analysis. All statistical results with p <0.05 were considered significant.

## Supplementary Material

Supplementary Tables
