# Supplementary material for: Retrospective study of gene signatures and prognostic value of m6A regulatory factor in non-small cell lung cancer using TCGA database and the verification of FTO
Source: Aging (Albany NY). 2020 Sep 9;12(17):17022–37. doi: 10.18632/aging.103622 (PMC7521517; doi:10.18632/aging.103622)
Supplement: Supplementary Tables [file aging-12-103622-s001..pdf]

SUPPLEMENTARY TABLES

Supplementary Table 1. Mutations of m6A regulatory genes in 408 cases.

sample	FTO	ALKBH5	YTHDF2	METTL14	WTAP	YTHDC1	YTHDC2	YTHDF1	METTL3
TCGA-05-4396-01	**1**	**1**	**1**				**1**	**1**	
TCGA-60-2725-01			**1**						
TCGA-17-Z008-01			**1**						
TCGA-49-4507-01			**1**						
TCGA-49-6742-01			**1**						
TCGA-50-6590-01			**1**						
TCGA-85-6560-01									**1**
TCGA-05-4417-01									**1**
TCGA-66-2788-01								**1**	
TCGA-05-4405-01								**1**	
TCGA-64-5775-01								**1**	
TCGA-22-1012-01									
TCGA-33-4538-01									
TCGA-66-2791-01									
TCGA-17-Z003-01									
TCGA-50-6594-01									
TCGA-73-4670-01									
TCGA-91-6836-01	**1**						**1**		
TCGA-18-3419-01							**1**		
TCGA-56-6545-01							**1**		
TCGA-66-2756-01							**1**		
TCGA-66-2763-01							**1**		
TCGA-17-Z022-01							**1**		
TCGA-35-3621-01							**1**		
TCGA-22-4591-01						**1**			
TCGA-33-6737-01						**1**			
TCGA-17-Z050-01						**1**			
TCGA-44-6775-01						**1**			
TCGA-50-6593-01						**1**			
TCGA-22-4595-01					**1**				
TCGA-66-2744-01					**1**				
TCGA-05-4420-01					**1**				
TCGA-73-4677-01					**1**				
TCGA-75-5125-01					**1**				
TCGA-75-6207-01					**1**				
TCGA-33-4566-01				**1**					
TCGA-05-4433-01				**1**					
TCGA-17-Z011-01				**1**					
TCGA-44-6777-01				**1**					
TCGA-50-5044-01				**1**					
TCGA-17-Z028-01		**1**							
TCGA-18-3407-01	**1**								
TCGA-22-5485-01	**1**								
TCGA-66-2766-01	**1**								
TCGA-05-4390-01	1								

The number 1 means there is a mutation of m6A regulatory genes in the sample.

Supplementary Table 2. Gene enrichment results of high expression of FTO in patients with NSCLC.

GS DETAILS	Size	ES	NES	Nom p-val	FDR q-val
HALLMARK_UV_RESPONSE_DN	136	-0.57	-1.96	0	0.069
HALLMARK_ANDROGEN_RESPONSE	96	-0.43	-1.81	0.018	0.12
HALLMARK_TGF_BETA_SIGNALING	54	-0.51	-1.76	0.025	0.12
HALLMARK_MYOGENESIS	199	-0.51	-1.57	0.011	0.22
HALLMARK_HEDGEHOG_SIGNALING	35	-0.58	-1.56	0.01996	0.21
HALLMARK_ANGIOGENESIS	36	-0.58	-1.55	0.034	0.19
HALLMARK_KRAS_SIGNALING_UP	194	-0.5222249	-1.5274777	0.02868852	0.2020273
HALLMARK_HEME_METABOLISM	191	-0.3403853	-1.5154847	0.01992032	0.1987541
HALLMARK_ESTROGEN_RESPONSE_EARLY	192	-0.3725932	-1.402012	0.04364326	0.23626567
